# Discovery of Natural Lead Compound from *Dendrobium* sp. against SARS-CoV-2 Infection

**DOI:** 10.3390/ph15050620

**Published:** 2022-05-18

**Authors:** Jutamas Jiaranaikulwanitch, Wipawadee Yooin, Nopporn Chutiwitoonchai, Worathat Thitikornpong, Boonchoo Sritularak, Pornchai Rojsitthisak, Opa Vajragupta

**Affiliations:** 1 Center of Excellence for Innovation in Analytical Science and Technology for a Biodiversity-Based Economic and Society (I-ANALY-S-T_B.BES-CMU), Chiang Mai University, Chiang Mai 50200, Thailand; jutamas.jia@cmu.ac.th (J.J.); wipawadee.y@cmu.ac.th (W.Y.); 2 Department of Pharmaceutical Sciences, Faculty of Pharmacy, Chiang Mai University, Chiang Mai 50200, Thailand; 3 Veterinary Health Innovation and Management Research Group, National Center for Genetic Engineering and Biotechnology (BIOTEC), National Science and Technology Development Agency (NSTDA), Bangkok 12120, Thailand; nopporn.chu@biotec.or.th; 4 Department of Food and Pharmaceutical Chemistry, Faculty of Pharmaceutical Sciences, Chulalongkorn University, Bangkok 10330, Thailand; worathat.t@pharm.chula.ac.th (W.T.); pornchai.r@chula.ac.th (P.R.); 5 Center of Excellence in Ageing and Chronic Diseases, Chulalongkorn University, Bangkok 10330, Thailand; boonchoo.sr@chula.ac.th; 6 Molecular Probes for Imaging Research Network, Faculty of Pharmaceutical Sciences, Chulalongkorn University, Bangkok 10330, Thailand; 7 Department of Pharmacognosy, Faculty of Pharmaceutical Sciences, Chulalongkorn University, Bangkok 10330, Thailand

**Keywords:** spike inhibitor, SARS-CoV-2, *Dendrobium*, antiviral infection, in silico screening

## Abstract

Since the pandemic of severe acute respiratory syndrome coronavirus (SARS-CoV-2) in December 2019, the infection cases have quickly increased by more than 511 million people. The long epidemic outbreak over 28 months has affected health and economies worldwide. An alternative medicine appears to be one choice to alleviate symptoms and reduce mortality during drug shortages. *Dendrobium* extract is one of the traditional medicines used for COVID-19 infection. Several compounds in *Dendrobium* sp. had been reported to exert pharmacological activities to treat common COVID-19-related symptoms. Herein, in silico screening of 83 compounds from *Dendrobium* sp. by using the SARS-CoV-2 spike protein receptor-binding domain (RBD) as a drug target was performed in searching for a new lead compound against SARS-CoV-2 infection. Four hit compounds showing good binding affinity were evaluated for antiviral infection activity. The new lead compound DB36, 5-methoxy-7-hydroxy-9,10-dihydro-1,4-phenanthrenequinone, was identified with the IC_50_ value of 6.87 ± 3.07 µM. The binding mode revealed that DB36 bound with the spike protein at the host receptor, angiotensin-converting enzyme 2 (ACE2) binding motif, resulted in antiviral activity. This study substantiated the use of *Dendrobium* extract for the treatment of SARS-CoV-2 infection and has identified new potential chemical scaffolds for further drug development of SARS-CoV-2 entry inhibitors.

## 1. Introduction

The pandemic of COVID-19 disease caused by severe acute respiratory syndrome coronavirus (SARS-CoV-2) has affected health and economies worldwide. By 3 May 2022, 511,965,711 confirmed cases of COVID-19 and 6,240,619 deaths had been reported by WHO since the first case was reported (WHO, https://covid19.who.int (accessed on 5 May 2022)). The number of infection cases is still increasing continuously. Due to the lack of effective and specific antiviral drugs against SARS-CoV-2, the discovery of new lead compounds is urgently needed. Among various natural products, medicinal herbs, the most frequently used for drug discovery, are the most essential and abundant sources of bioactive compounds [1]. *Dendrobium* sp. is a plant that has been used in traditional Chinese medicine (TCM) for thousands of years to nourish Yin, increasing the production of body fluids and heat-clearing [2,3]. “Shihu” is the common name of thirty *Dendrobium* species such as *D. nobile*, *D. chrysotoxum*, *D. fimbriatum* and others [4]. Another common name of TCM from *D. officinale* is “Tiepi shihu” [3]. The monographs of both Shihu and Tiepi shihu were officially listed in Chinese Pharmacopoeia for authentication and quality assessment [3]. The previous prescreening biochemical analysis data showed the presence of alkaloids [5] and other compounds such as bibenzyls, phenanthrenes, polysaccharides, fluorenones and flavonoids in *Dendrobium* sp. [6]. Other phenolic acids and organic acids were also identified in *Dendrobium* sp. [7,8]. The reported pharmacological activities of *Dendrobium* species were diverse, including hepatic lipid and gluconeogenesis regulation [5], antioxidant [9], anti-inflammatory [5,9,10], antifungal [9,11], antimicrobial [9,12], anticancer [4,5,13], antimutagenic [9], antidiabetic [4,5,9], neuroprotective [4,5,9], immunomodulatory [4,9], antiplatelet aggregation [9] and antiviral [5,14]. Moreover, *Dendrobium nobile* is one of the TCM used to treat COVID-19 infection in northern China [15]. Thus, *Dendrobium* species is a potential plant for the lead discovery of drugs against SARS-CoV-2.

Various approaches for anti-SARS-CoV-2 drug discovery have been employed by blocking viral drug targets including viral entry, viral enzymes, viral replication, and viral translation and release [2]. Spike glycoprotein on the surface of SARS-CoV-2 is a potential drug target due to its crucial role in the first infection step. The S1 receptor-binding subunit and S2 membrane fusion subunit in the spike (S) protein are identified as viral proteins mediating virus entry into host cells [16]. The S1 subunit of the spike protein contains a receptor-binding domain (RBD) that recognizes and binds the human receptor angiotensin-converting enzyme 2 (hACE2). After the binding of the RBD in the S1 subunit to the hACE2, the next step is the S2 subunit-mediated membrane fusion of the viral envelope with the host-cell membrane [17]. The S1 subunit consists of the *N*-terminal domain (NTD, 14–305 aa), receptor-binding domain (RBD, 319–541 aa) and receptor binding motif (RBM, 437–508 aa) [18]. The RBM in the S1 subunit is crucial for the ACE2 binding as the amino acid residues in the RBM region contribute to the binding interactions of spike protein with the ACE2 receptor [19]. The SARS-CoV-2 spike protein, particularly the S1 subunit, has been used as a drug target in searching for anti-COVID-19 drugs. Several in silico screenings against the SARS-CoV-2 spike protein have been reported, but most used a compound library of existing drugs aimed at drug repurposing [20]. Herein, in silico screening of 83 compounds from *Dendrobium* sp. against the SARS-CoV-2 spike protein was performed and the lead compound, DB36, with promising antiviral infection, was identified.

## 2. Results

### 2.1. In Silico Screening Study

**Figure 1:**
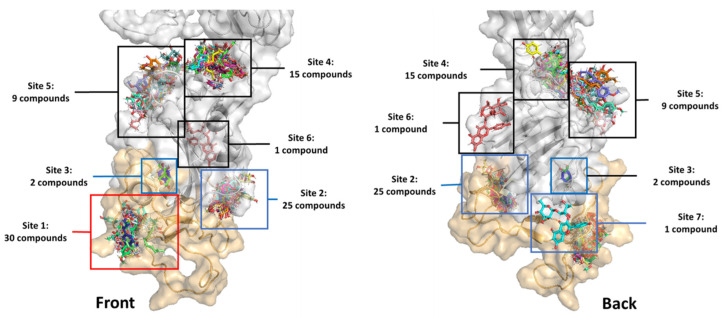
The binding sites of 83 in-house library compounds from *Dendrobium* sp. The orange surface is the receptor-binding motif (RBM) of amino acid residues 437–508.

The in-house 83 compounds from *Dendrobium* sp. were compiled to build up a focus *Dendrobium* compound library. The chemotypes of compounds in the *Dendrobium* library are diverse, including flavonoid, flavonoid glycosides, phenolics, bibenzyls and bis(bibenzyls), phenanthrenes, bibenzyl-dihydrophenanthrenes, lignans, furanones, coumarins, chromones, acid and triterpenoids. These collected compounds were extracted from *Dendrobium* sp., including *D. draconis, D. formosum, D. brymerianum, D. palpebrae, D. venustum, D. scabrilingue, D. parishii, D. parishii, D. ellipsophyllum, D. falconeri, D. pulchellum, D. tortile* and *D. capillipes*. All compounds were docked with the spike protein subunit S1 (PDB code: 6VYB). The lowest binding free energy conformation (∆G_binding_) of the highest cluster was ranked and evaluated for the binding mode. The docking result revealed that all compounds bound with the S1-spike protein in seven different sites, as shown in Figure 1, with ∆G_binding_ in a range of −11.89 to −4.06 Kcal/mol. Thirty compounds were found to interact with the amino acid residues at the receptor-binding motif (ACE2-RBM) of the receptor-binding domain (RBD). While twenty-five, two and one compounds are bound outside the ACE2-RBM at sites two, three and seven, respectively. However, these three binding sites were adjacent to the RBM and the binding may induce the conformational changes of RBM to be unable to bind to the ACE2 receptor. Other compounds bound to sites four, five and six outside the RBD.

**Figure 2:**
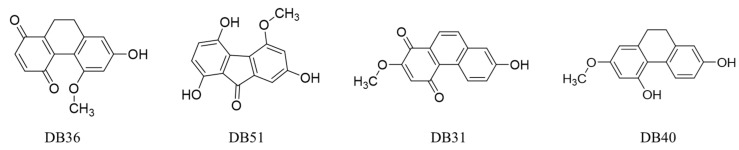
The chemical structures of hit compounds arising from the virtual screening.

The binding energy of all compounds was ranked by the free energy of binding. The top eight compounds with binding energy lower than −6.00 Kcal/mol, with the cluster over 35 conformations are shown in Table 1. The top four hit compounds (DB36, DB51, DB31 and DB40) bound at sites one and two were selected for the antivirus infectivity assay. The chemotype of these hits consists of dihydrophenanthrenes, fluorenones and phenanthrenes. The chemical structure of the four hit compounds are shown in Figure 2.

### 2.2. In Vitro Verifying Antiviral Activity of Hit Compounds against SARS-CoV-2 S–hACE2 Mediated Virus Infection

The antiviral activity against SARS-CoV-2 of hit compounds was evaluated by the in vitro assay model. The SARS-CoV-2 S pseudotyped HIV (carrying luciferase reporter gene) was used as a surrogate SARS-CoV-2 and human ACE2-TMPRSS2 expressing cells (hTMPRSS2 transfected HEK293T/17-hACE2 cells) as the host cells. The spike protein was used to bind with the ACE2 receptor on human host cells to initiate the infection process. After the SARS-CoV-2 S pseudotyped HIV infected the host cells, the luciferase substrate was catalyzed by the luciferase reporter enzyme resulting in luminescence. Thus, the luminescent signal directly correlates with the amount of viruses infecting or entering the host cell.

**Figure 3:**
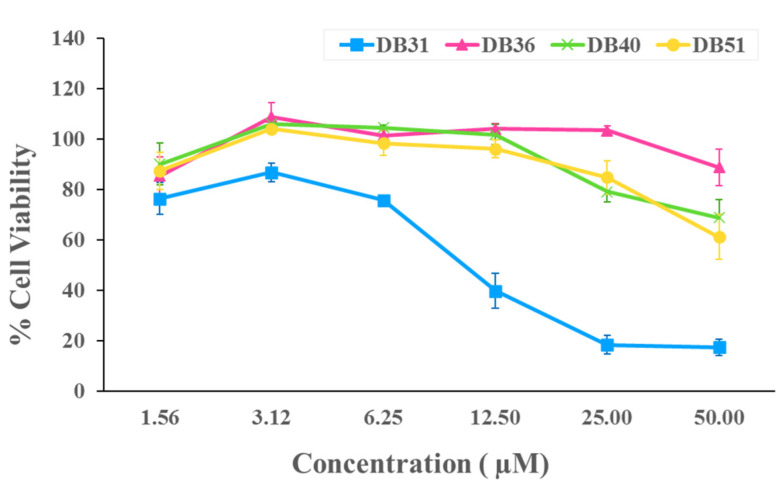
Cytotoxicity of identified hit compounds from in silico screening. HEK293T/17-hACE2 cells were treated with DB31, DB36, DB40, and DB51 at concentrations of 1.56, 3.12, 6.25, 12.50, 25 and 50 μM for 48 h. Cell viability was determined by Cell Counting Kit-8 solution at the optical density of 450 nm. Percent cell viability was calculated by comparing with DMSO. Data are expressed as the relative mean value with an error bar (standard error of the mean, SEM) from three independent experiments (each performed in triplicate).

For the infectivity assay, the non-cytotoxic concentrations of four selected hit compounds were first determined by the cytotoxicity test at 1.56, 3.12, 6.25, 12.50, 25 and 50 μM. Compound DB36 showed the lowest toxicity, followed by DB40, DB51 and DB31 (Figure 3). The non-cytotoxic concentrations of each compound were used in the initial screening for antiviral infection at a concentration of 12.50 μM, except compound DB31, which was tested at a non-toxic concentration of 3.12 μM.

**Figure 4:**
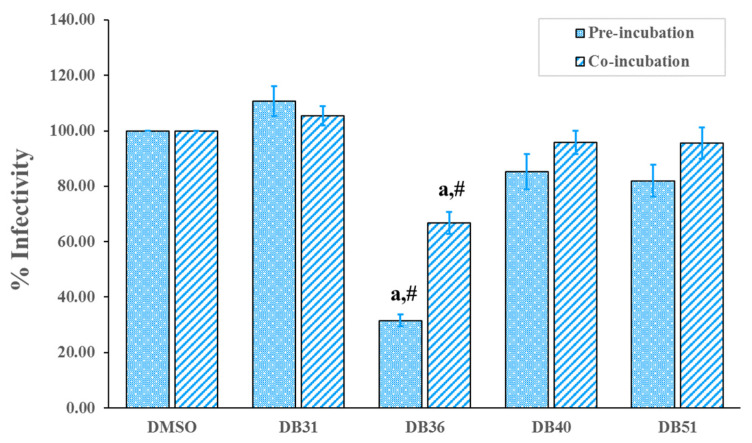
Screening of hit compounds against pseudovirion (SARS-CoV-2 S pseudotyped HIV) infection to host cells, HEK293T, at a concentration of 12.50 μM with the exception of DB31 at a concentration of 3.12 μM. Confluent HEK293T/17-hACE2 cells overexpressing hTMPRSS2 were incubated with pseudovirion and hit compounds in pre-incubation conditions (virus and the hit compound incubated for one hour before infected to the cell) or co-incubation (virus and the hit compound immediately infected to the cells). BS-R2B2 (MonoRab SARS-CoV-2 neutralizing antibody) at 1 µg/mL was used as a positive control. The percentage of infectivity of each bar presented the percentage of SARS-CoV-2 S-hACE2 mediated infectivity as mean ± SEM (*n* = 3). The letter ‘a’ denotes a significant difference compared with the control DMSO (*p* < 0.05), and the symbol ‘#’ denotes a significant difference between pre- and co-incubation of each compound, based on one-way ANOVA using GraphPad Prism 5.02.

Antivirus infection activity of hit compounds was determined in the pre-incubation and co-incubation protocols. For pre-incubation, SARS-CoV-2 S pseudotyped HIV was incubated with the hit compound for one hour before being added to the host cells. While co-incubation, SARS-CoV-2 S pseudotyped HIV and hit compound were immediately added to the host cells. After incubation with host cells for 48 h, luminescence was determined. The results showed that compound DB36, 5-methoxy-7-hydroxy-9,10-dihydro-1,4-phenanthrenequinone, at a concentration of 12.5 µM, was able to reduce SARS-CoV-2 S pseudotyped HIV infectivity significantly compared to the control group, 0.5% DMSO, in both conditions of pre-incubation and co-incubation. In contrast, other hit compounds tested at the same concentration as compounds DB40 and DB51 and the lower concentration as compound DB31 had not reduced infection (Figure 4). In the case of co-incubation, compound DB36 had lower activity than pre-incubation, significantly (Figure 4).

**Figure 5:**
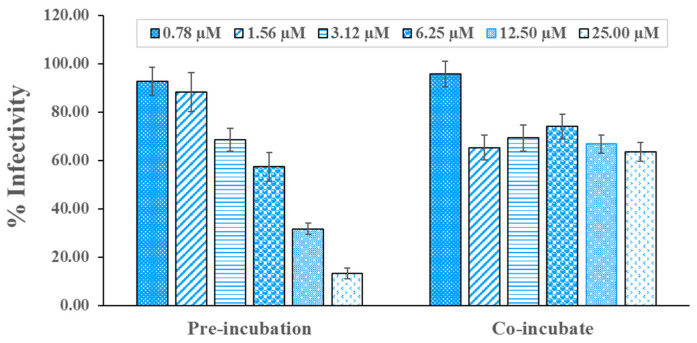
DB36 inhibits SARS-CoV-2 S-hACE2 mediated virus entry in a dose-dependent manner. SARS-CoV-2 S pseudotyped HIV was pre-incubated with DB36 at a concentration of 0.78, 1.56, 3.12, 6.25, 12.50, and 25.00 µM for one hour and infected to HEK293T/17-hACE2 overexpressing hTMPRSS2 (pre-incubation) or immediately infected to the cells (co-incubation). Infectivity of the virus was determined by measuring the activity of expressed luciferase reporter gene. Data are expressed as the relative mean value with an error bar (SEM) from 2–3 independent experiments (each performed in triplicate).

Thus, compound DB36 was selected to determine the antiviral infection activity in a dose-response relationship for the estimation of the half-maximal inhibitory concentration (IC_50_) value. Interestingly, compound DB36 is effective against SARS-CoV-2 S infection in a dose-dependent manner in the pre-incubation condition. In contrast, the maximum inhibition of DB36 in co-incubation was only 35% at a concentration of 1.56 µM (Figure 5). In pre-incubation, the IC_50_ values against viral infection of DB36 were 6.87 µM, and the half-maximal cytotoxic concentration (CC_50_) was more than 50 µM, thus yielding a satisfactory selectivity index (SI) of more than 7.28 (Table 2). This hit compound showed antiviral infection at a concentration level of micromolar comparable to the reported repurposing drugs for SARS-CoV-2, such as hydroxychloroquine and sertraline [21].

### 2.3. DB36 Directly Inhibits SARS-CoV-2 S–hACE2 Binding

**Figure 6:**
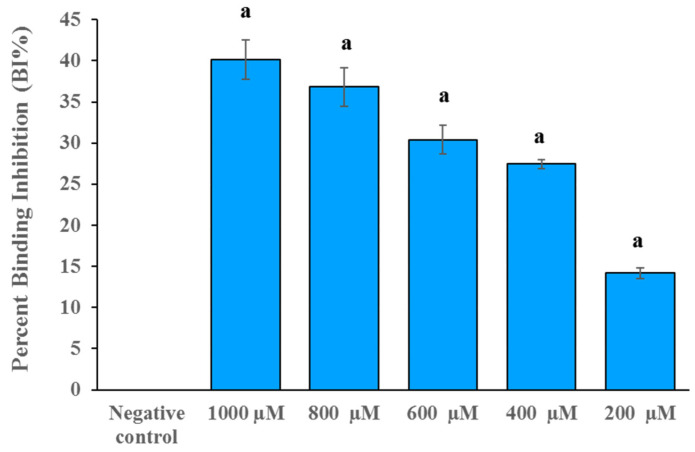
The spike RBD and ACE2 binding inhibition of DB36 in a dose-dependent manner. Data are expressed as the relative mean value with an error bar (SEM) from three independent experiments (each performed in duplicate). BS-R2B2 (MonoRab SARS-CoV-2 neutralizing antibody) at 1 µg/mL was used as a positive control. The letter ‘a’ denotes a significant difference compared with the negative control (*p* < 0.05) based on one-way ANOVA using GraphPad Prism 5.02.

The insight-mediated mechanism for antivirus infectivity of compound DB36 was further investigated. The SARS-CoV-2 S–hACE2 binding assay was conducted to determine the effect of DB36 on the entry of SARS-CoV-2 into host cells at a molecular level. The result revealed that compound DB36 could inhibit the binding between the SARS-CoV-2 spike (RBD) protein and ACE2 receptor in a dose-dependent manner (Figure 6). The percent binding inhibition was 14.19 to 40.16% at a concentration of 200 to 1000 µM. The result suggested that the inhibition of SARS-CoV-2 spike–ACE2 binding mediated the antiviral infection activity of the hit compound DB36.

## 3. Discussion

**Figure 7:**
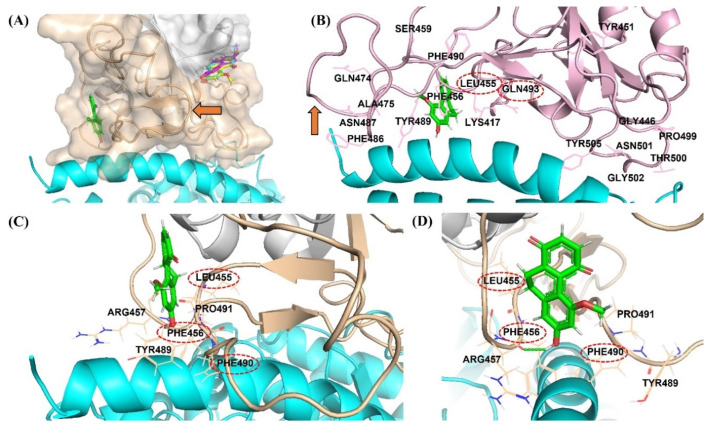
Binding mode of DB36 (green color stick) located in the binding motif (RBM) region (orange color surface) at the interface area of the spike protein interacting with ACE2 receptor (cyan helix color): (**A**) Binding location of DB36 at RBD of apo conformation of spike protein comparing with other hits; (**B**) The superimposition of the docked pose of DB36 with the ACE2 bound conformation of the spike protein (PDB code: 7KMB, pink color); The red arrow showed the flexible loop three (residues 472–490) of the spike protein in conformations of (**A**) apo (orange color) and (**B**) ACE2 bound (pink color); (**C**,**D**) The interactions of DB36 with the residues at the interface area of the spike protein interacting with the ACE2 receptor.

The antivirus infectivity and spike binding assay results are in good agreement with the binding modes from the in silico experiment. The binding modes of hit compounds revealed that compound DB36 is fully bound with the amino acid residues in the spike protein at the RBM region. In contrast, other hit compounds are partially bound with amino acid residues at RBM of apo conformation spike protein (Figure 7A). The binding of DB36 at the chain–chain interface of the spike RBM-ACE2 complex plays an essential role in binding and stabilizing the complex structure of the virus spike protein and the ACE2 receptor of host cells, the first step of host entry [22]. DB36 interacted with the key amino acid residues in the RBM of the spike protein, namely Leu455, Phe456 and Phe490 (Figure 7C,D). Since these residues of the virus spike protein participated in H-bond formation with the ACE2 receptor for host entry, the binding of these residues by DB36 resulted in the inhibition of viruses binding with ACE2 and entering the host cells. The reported key amino acid residues of SARS-CoV-2 S-RBM providing H-bond interactions with ACE2 receptor were Leu455, Phe456, Ala475, Phe486, Phe490 and Gln493 and the amino acid residues for stabilizing the complex structure were Ser459, Gln474, and Pro499 [23]. The mutagenesis of these key amino acid residues resulted in a decrease in the binding affinity with the ACE2 receptor [23]. Notably, the amino acid residues Leu455 and Gln493 of the spike protein are crucial for forming hydrogen bonds with amino acid residues Lys31 and Glu35 of the ACE2 receptor [24].

Moreover, the located DB36 at RBM possibly obstructs or restricts the flexibility loop three of the spike protein to accommodate and stabilize the bound conformation of spike protein (Figure 7A,B). The three amino acids within flexible loop three including Phe486, Asn487 and Tyr489 were reported as stabilizing residues interacting with ACE2 (Figure 7B) [25,26]. This hinder of DB36 reduces the stabilization of bound conformation of spike protein and ACE2, leading to greater antiviral infectivity of pre-incubation over co-incubation. The binding at this flexible loop three was also found in some neutralizing antibodies designed to bind with RBD such as CC12.1 [27], P2C-1F11 [28], and S2E12 [29].

**Figure 8:**
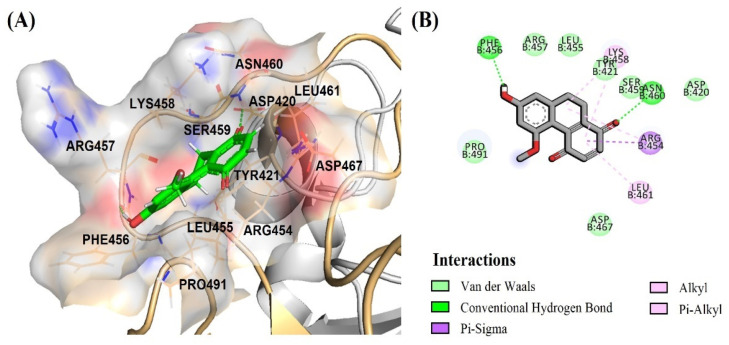
Binding mode of DB36 (green color) located in the binding motif (RBM) region: (**A**) surface view of DB36 binding mode; (**B**) amino acid interactions of DB36.

**Figure 9:**
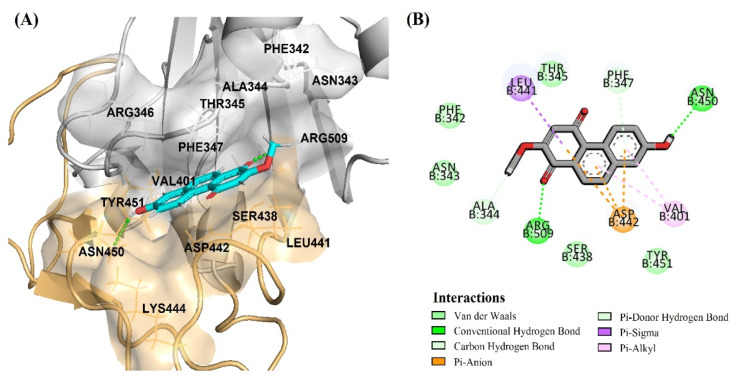
Binding modes of DB31 located in the S1-RBD of the spike protein adjacent to the binding motif (RBM) region in an orange color surface: (**A**) surface view of DB31 binding position between RBM (orange color) and RBD (gray color) regions; (**B**) amino acid interactions of DB31.

Thus, the most significant antivirus infectivity of DB36 among other hit compounds is possibly due to the fully bound in the RBM region and had interactions with the key residues Phe456 via a hydrogen bond and Leu455 and Ser459 via van der Waals (Figure 8A,B). Meanwhile, other hits DB31, DB40 and DB51 were partially bound with the RBM region of the spike protein and did not interact with key amino acids in the RBM region. The amino acid in the RBM region, in which DB31, DB40 and DB51 interacted, were Ser438, Leu441, Asp442, Lys444, Asn450, and/or Tyr451 (Figure 9 and Figure S1).

Although the inhibition results of DB36 on virus infectivity and the SARS-CoV-2 S–hACE2 binding appeared to be promising, structural optimization is needed to improve the potency. To increase the antivirus infection via anti-spike protein binding, the expansion of the binding area for more interactions with key residues at the binding interface of spike protein should be further carried out.

## 4. Materials and Methods

### 4.1. General

Molecular dockings were performed using AutoDockTools-1.5.6. All compounds were generated by Ultra 13.0 and ChemBio3D Ultra 13.0 and optimized using Gaussian 09 program. PyMOL program and Discovery Studio were used to create docking visualization and docking interaction. Microsoft Excel 2013 and GraphPad Prism version 5.02 were used to generate graph visualization and calculate IC_50_ values. Statistical analysis of cell viability for the antivirus infection activity study was conducted by one-way ANOVA using GraphPad Prism version 5.02.

### 4.2. In Silico Screening Study

#### 4.2.1. Data Collection and Ligand Preparation

The structures of 83 compounds from *Dendrobium* sp. reported by our group (Supplement Table S1) were collected to construct an in-house library in 2D and 3D using ChemBioDraw and ChemBio3D. The ligands were optimized using the Gaussian 09 program with the B3LYP/6-31G (d, p) method. Then, Gasteiger’s charges were assigned using AutoDockTool-1.5.6.

#### 4.2.2. Spike Protein Template Preparation

The spike protein of SARS-CoV-2 (PDB code: 6VYB) at 3.20 Å resolution was selected from the RCSB Protein Data Bank [30]. The open state domain of the trimer was separated to prepare a template. Water molecules were removed from the structure, and missing amino acid residues and hydrogen atoms were added to the system by BIOVIA Discovery Studio 2017 R2 Client. Gasteiger’s charges were assigned using AutoDockTool-1.5.6. A grid box was generated to cover the binding site and allosteric site of the S1 receptor-binding domain (amino acid residues 319–591) [31] using AutoGrid 4.0. The size of the grid was set as 126 × 100 × 100 Å^3^ with a grid spacing of 0.375 Å based on center (X, Y, Z) of 107.285, 180.579 and 208.827, respectively.

#### 4.2.3. Docking and Interaction Visualization

The preparation ligands were docked with the spike protein template using AutoDockTool-1.5.6. The docking parameters were as follows: 100 runs of the GA, a population size of 300, energy evaluations per run of 2,500,000 and a maximum number of generations of 27,000. The best binding energy of the highest cluster of each compound was ranked. The binding interactions of ligand and the spike protein were visualized using PyMOL and Discovery Studio.

### 4.3. In Vitro Verification Assay

#### 4.3.1. Preparation of Test Compounds

The test compounds, DB31 [32], DB36 [33,34], DB40 [32] and DB51 [35] were dissolved in dimethyl sulfoxide (DMSO), and diluted with DMSO to a desired concentration for in vitro assay.

#### 4.3.2. Cell Lines and Pseudotyped Virus

Human embryonic kidney (HEK)293T/17 cells (ATCC: CRL11268) stably expressing a SARS-CoV-2 entry receptor of human cells, hACE2 (HEK293T/17-hACE2) were generated by lentivirus transduction as described previously [36,37] and maintained in Dulbecco’s Modified Eagle Medium/high glucose (Hyclone Laboratories, Logan, UT, USA) supplemented with 10% (*v*/*v*) fetal bovine serum and antibiotics [36,38].

The HIV-based lentivirus carrying a firefly luciferase reporter gene was pseudotyped with SARS-CoV-2 S protein (SARS-CoV-2 S pseudotyped HIV) by the method mentioned previously [36,39]. Briefly, the infectious viral particles were generated by co-transfection of pCSFLW, pCMV-ΔR8.91, and the pCAGGS plasmid carrying codon-optimized SARS-CoV-2 S gene (GenBank: YP_009724390.1) into HEK293T/17 cells. The supernatant containing SARS-CoV-2 S pseudotyped viral particles was harvested and stored at −80 °C until used.

#### 4.3.3. Cytotoxicity Assay

HEK293T/17-hACE2 cells were seeded in a 96-well tissue culture microplate (4 × 10^4^ cells/well) and incubated in a 5% CO_2_ incubator at 37 °C overnight. Each test compound was diluted with DMSO and added to the cells at the final concentrations of 1.56, 3.12, 6.25, 12.50, 25 and 50 µM. The final concentration of DMSO was normalized to 0.5% *v*/*v* to minimize the cytotoxic effect. DMSO-treated cells were used as a control to indicate 100% cell viability. After 48 h incubation, cytotoxicity was determined by adding Cell Counting Kit-8 reagent (Dojindo, Rockville, MD, USA) to the cells followed by incubation in a 5% CO_2_ incubator at 37 °C for 1 h. Then, the absorbance at 450 nm was measured by Synergy HTX Multi-Mode Microplate Reader (BioTek, Winooski, VT, USA). The percentage of cell viability was calculated by comparing the absorbance value of the test compound with DMSO as a control.

#### 4.3.4. SARS-CoV-2 S–hACE2 Mediated Infectivity Assay

The SARS-CoV-2 S–hACE2 mediated virus entry system based on the neutralization assay [36,38] was used to determine the inhibitory effect of test compounds by infecting SARS-CoV-2 S pseudotyped HIV into the host cells, HEK293T/17-hACE2 cells overexpressing the human transmembrane serine protease 2 (hTMPRSS2), which are required for virus entry by fusion [40]. Briefly, the HEK293T/17-hACE2 cells (4.5 × 10^6^ cells) were seeded in a 10 cm tissue culture dish overnight and transfected with 150 ng of pCAGGS plasmid carrying hTMPRSS2 gene (GenBank: BC051839.1) using FuGENE HD Transfection Reagent (Promega, Madison, WI, USA) for 24 h to allow the expression of hTMPRSS2. The pre-incubation protocol was performed by incubating 50 µL of SARS-CoV-2 S pseudotyped HIV (at 1 × 10^4^ relative luminescence units) with 50 µL of medium containing test compound (at the non-cytotoxic concentration) in an opaque 96-well white microplate (PerkinElmer, Waltham, MA, USA) at 37 °C for 1 h. Then, the HEK293T/17-hACE2 cells overexpressing hTMPRSS2 were trypsinized and resuspended in a fresh medium at a density of 1 × 10^6^ cells/mL. Fifty microliters of the cell suspension were added to the compound-treated virus. The cells were incubated in a 5% CO_2_ incubator at 37 °C for 48 h to allow infection and the luciferase reporter gene expression. The DMSO-treated SARS-CoV-2 S pseudotyped HIV was used as a control to define 100% infectivity and the monoclonal antibody against SARS-CoV-2 receptor-binding domain (RBD). MonoRab SARS-CoV-2 Neutralizing Antibody (BS-R2B2) (GenScript, Piscataway NJ, USA) was used as a positive control. Then, luciferase activity was determined using Bright-Glo Luciferase Assay System (Promega) by removing the culture medium and adding 25 µL of PBS-diluted luciferase assay substrate (ratio 1:1) to the infected cells and incubating the cells at room temperature for 5 min. The luminescence signal was measured by Synergy HTX Multi-Mode Microplate Reader (BioTek). The percentage of SARS-CoV-2 S-hACE2 mediated infectivity was calculated by comparing the luciferase activity of the test compound with DMSO as a control. The co-incubation protocol was performed by adding HEK293T/17-hACE2 cells overexpressing hTMPRSS2 into the mixture of the test compound and SARS-CoV-2 S pseudotyped HIV without pre-incubation for 1 h.

#### 4.3.5. SARS-CoV-2 S–hACE2 Binding Inhibition Assay

The direct inhibitory effect of test compounds on SARS-CoV-2 S–hACE2 binding was determined by ELISA using the SARS-CoV-2 S RBD-coated 96-well strip microplate from the COVID-19 Spike-ACE2 binding assay kit (RayBiotech, Peachtree Corners, GA, USA). Briefly, the 1x ACE2 Protein was mixed with the test compound at different concentrations (by normalizing the final concentration of DMSO solvent to 5% *v*/*v*) and added to the spike RBD-coated plate for 2.5 h incubation at room temperature. The COVID-19 Spike-ACE2 Binding Assay Kit Controls (RayBiotech), which are the purified antibodies that do not inhibit or inhibit SARS-CoV-2 S–ACE2 binding, were used as negative and positive controls, respectively. The strip microplate was washed with 1x Wash Solution, incubated with 1x Detection Antibody followed by incubation with 1x HRP-Conjugated Anti-IgG, TMB substrate, and stop solution according to the manufacturer’s protocol. The absorbance at 450 nm was measured using Synergy HTX Multi-Mode Microplate Reader (BioTek). The percentage of SARS-CoV-2 S–hACE2 binding was calculated by comparing the absorbance value of the test compound with a negative control.

## 5. Conclusions

The in silico study of the SARS-CoV-2 spike protein can be used as a screening tool to identify a new lead compound to treat COVID-19. The new lead compound DB36, 5-methoxy-7-hydroxy-9,10-dihydro-1,4-phenanthrenequinone, found in *D. draconis* [33] and *D. formosum* [34] was identified to inhibit virus infection with an IC_50_ value of 6.87 µM. The mechanism mediated antivirus infection appeared to inhibit the SARS-CoV-2 spike protein (RBD) from binding to the ACE2 receptor. The in silico binding study suggested that the binding interactions with the residues in the RBM region of the spike S1 subunit, particularly the interface area, play an essential role in antivirus infectivity. The discovery of antiviral infection activity of DB36 and its mediated mechanism supports the use of *D. draconis* and *D. formosum* for COVID-19 treatment. Similar to DB36, docking results revealed that *p*-hydroxybenzoic acid and (-)-shikimic acid bound with comparable binding energy at the same binding site. In summary, this is the first report showing the potential of both compounds against SARS-CoV-2 infection. Structural modification of the two compounds is needed to optimize binding affinity and enhance antiviral activity.

## Figures and Tables

**Table 1:** Docking result of the top-ranking compounds from *Dendrobium* sp. against the SARS-CoV-2 spike protein.

Code	Number in Cluster	∆G_binding_ (Kcal/mol)	Site Number	Compound	Chemotype
DB36	35	−8.09	1	5-Methoxy-7-hydroxy-9,10-dihydro-1,4-phenanthrenequinone	Dihydro phenanthrenes
DB51	68	−7.85	2	Dendroflorin	Fluorenones
DB31	75	−7.74	2	Densiflorol B	Phenanthrenes
DB40	44	−6.92	2	Lusianthridin	Dihydro phenanthrenes
DB50	51	−6.7	2	Nobilone	Fluorenones
DB30	44	−6.69	2	Flavanthrinin	Phenanthrenes
DB65	36	−6.15	1	p-Hydroxybenzoic acid	Phenolics
DB74	38	−6.06	1	(-)-Shikimic acid	Acids

**Table 2:** Antiviral infection activity of hit compounds.

Compounds	Concentration (µM)	% Infection ± SEM	IC_50_ (µM)	CC_50_ (µM)	SI (CC_50_/IC_50_)
DB31	3.12	110.81 ± 5.41	-	11.85	-
DB36	12.50	31.57 ± 2.37	6.87	>50	>7.28
DB40	12.50	85.23 ± 6.37	-	>50	-
DB51	12.50	81.98 ± 5.83	-	>50	-
Hydroxychloroquine	-	-	1.33 ^a^	>30 ^a^	>22.56 ^a^
Sertraline	-	-	9.34 ^a^	27.84 ^a^	2.98 ^a^

^a^ Ref: [21].

## Data Availability

Not applicable.

## Supplementary Materials

The following supporting information can be downloaded at: https://www.mdpi.com/article/10.3390/ph15050620/s1, Table S1: The structures of 83 compounds from *Dendrobium* sp. docked against SARS-CoV-2 spike protein; Figure S1: Binding modes of DB31, DB40 and DB51 located in the S1-RBD of spike protein adjacent to the binding motif (RBM) region as shown in an orange color.
