# Supplementary material for: Discovery of Natural Lead Compound from Dendrobium sp. against SARS-CoV-2 Infection
Source: Pharmaceuticals (Basel). 2022 May 18;15(5):620. doi: 10.3390/ph15050620 (PMC9143658; doi:10.3390/ph15050620)
Supplement: Supplementary file 1 [file pharmaceuticals-15-00620-s001.zip › pharmaceuticals-1694518-supplementary.pdf]

2.1. *In silico* Screening Study

The structure of 83 compounds found in Dendrobium sp. were collected to construct in-house library in 2D and 3D using ChemBioDraw and ChemBio3D.

Table S1. The structures of 83 compounds from Dendrobium sp. docked against SARS-CoV-2 spike protein

Code	Compound	Structure
DB01	(2*S*)-Homoeriodictrol	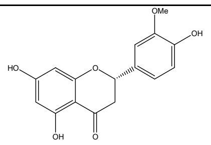
DB02	(2*S*)-Eriodictyol	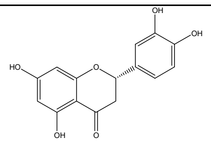
DB03	Quercetin-3-O-α-L-rhamnopyranosyl-(1→2)-β-D-xylopyranoside	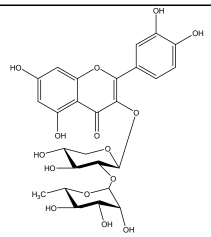
DB04	Kaempferol-3-O-α-L-rhamnopyranosyl-(1→2)-β-D-xylopyranoside	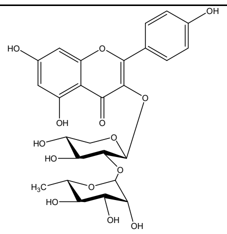
DB05	Kaempferol-3-O-α-L-rhamnopyranosyl-(1→2)-β-D-glucopyranoside	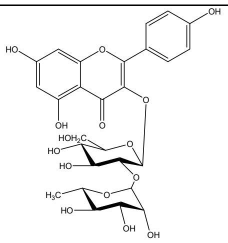
DB06	Kaempferol-3,7-O-di-α-L-rhamnopyranoside	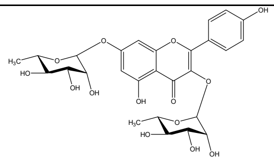

Table S1. The structures of 83 compounds from Dendrobium sp. docked against SARS-CoV-2 spike protein (cont.)

Code	Compound	Structure
DB07	Quercetin-3-O-α-L-rhamnopyranoside	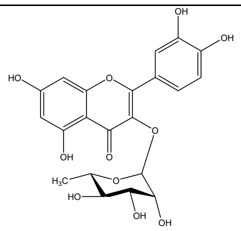
DB08	Kaempferol-3-O-α-L-rhamnopyranoside	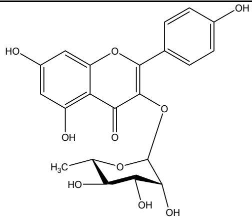
DB09	Gigantol	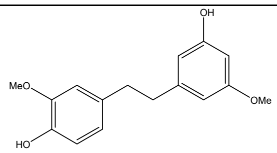
DB10	Batatasin III	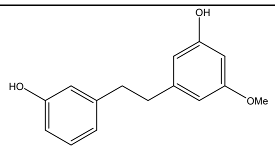
DB11	4,5,4′-Trihydroxy-3,3′-dimethoxybibenzyl	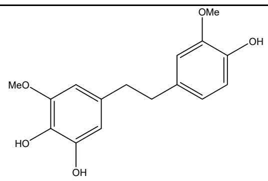
DB12	Brittonin A	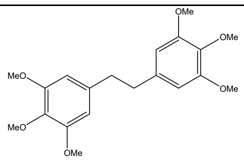
DB13	Moscatilin<br>(Dendrophenol)	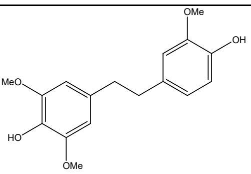
DB14	Chrysotobibenzyl	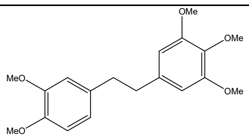

Table S1. The structures of 83 compounds from Dendrobium sp. docked against SARS-CoV-2 spike protein (cont.)

Code	Compound	Structure
DB15	Crepidatin	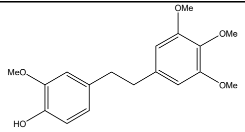
DB16	Chrysotoxine	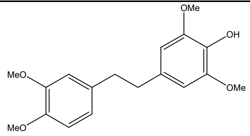
DB17	5-hydroxy-3,4,3′,4′,5′-pentamethoxybibenzyl	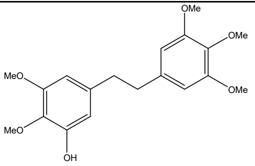
DB18	4,4′-Dihydroxy-3,5-dimethoxybibenzyl	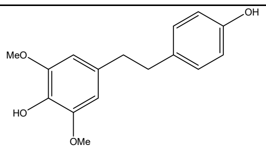
DB19	Tristin	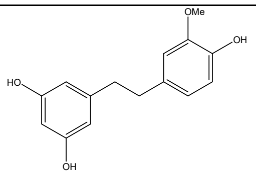
DB20	3,4-Dihydroxy-3,4′-dimethoxybibenzyl	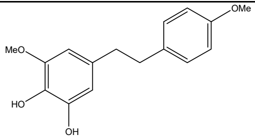
DB21	Dendrocandin B	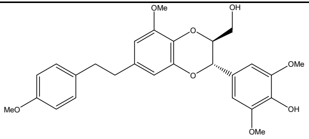
DB22	Dendrocandin E	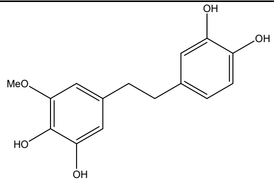
DB23	Dendrocandin I	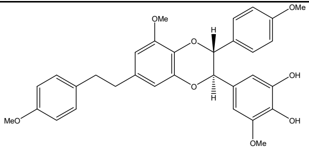

Table S1. The structures of 83 compounds from Dendrobium sp. docked against SARS-CoV-2 spike protein (cont.)

Code	Compound	Structure
DB24	4,3′,4′-Trihydroxy-3,5-dimethoxybibenzyl	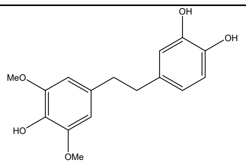
DB25	Aloifol I	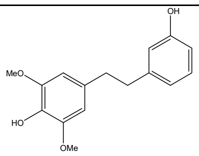
DB26	3,3′-Dihydroxy-<br>4,5-dimethoxybibenzyl	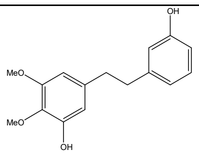
DB27	5,4′-dihydroxy-3,4,3′-trimethoxybibenzyl	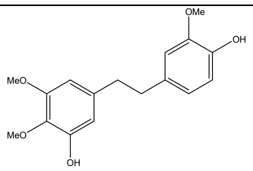
DB28	Dendrosinen B	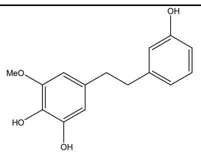
DB29	Fimbriatone	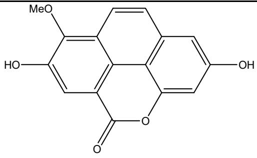
DB30	Flavanthrinin	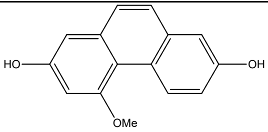
DB31	Densiflorol B	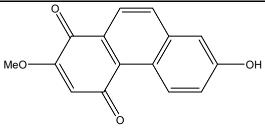
DB32	Confusarin	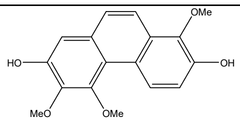
DB33	Nudol	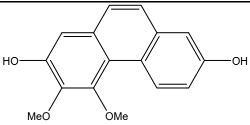

Table S1. The structures of 83 compounds from Dendrobium sp. docked against SARS-CoV-2 spike protein (cont.)

Code	Compound	Structure
DB34	2,5-Dihydroxy-4,9-dimethoxyphenanthrene	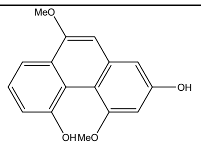
DB35	Dendroscabrol A	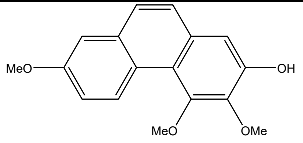
DB36	5-Methoxy-7-hydroxy-9,10-dihydro-1,4-phenanthrenequinone	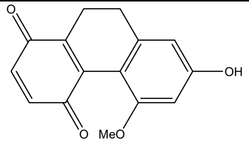
DB37	Hircinol	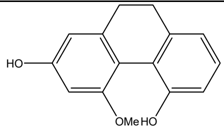
DB38	7-Methoxy-9,10-dihydro-phenanthrene-2,4,5-triol	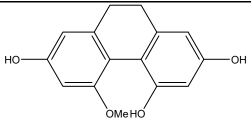
DB39	4,5-Dihydroxy-2,3-dimethoxy-9,10-dihydrophenanthrene	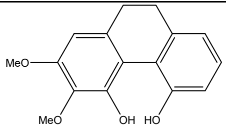
DB40	Lusianthridin	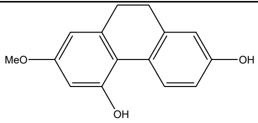
DB41	Erianthridin	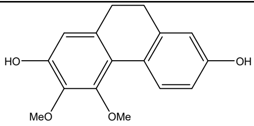
DB42	Coelonin	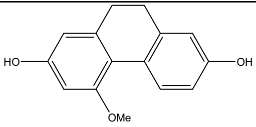
DB43	2,5,7-Trihydroxy-4-methoxy-9,10-dihydrophenanthrene	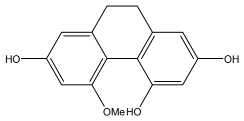

Table S1. The structures of 83 compounds from Dendrobium sp. docked against SARS-CoV-2 spike protein (cont.)

Code	Compound	Structure
DB44	Dendroinfundin A	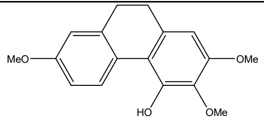
DB45	Dendroinfundin B	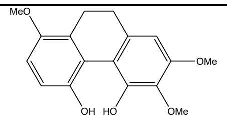
DB46	Ephemeranthol A	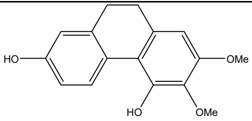
DB47	Syringaresinol	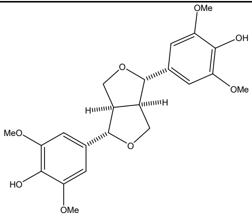
DB48	Liriodendrin	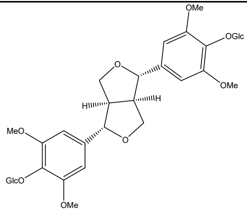
DB49	4-(2-Hydroxypropyl)-2(*5H*)-furanone	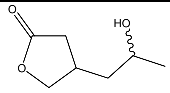
DB50	Nobilone	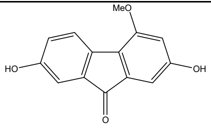
DB51	Dendroflorin	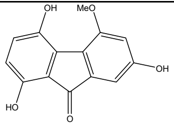
DB52	Denchrysan B	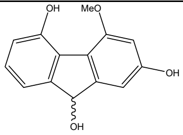
DB53	Scoparone	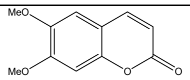

Table S1. The structures of 83 compounds from Dendrobium sp. docked against SARS-CoV-2 spike protein (cont.)

Code	Compound	Structure
DB54	5,7-Dihydroxy-chromen-4-one (5,7-Dihydroxychromone)	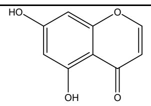
DB55	Asiatic acid	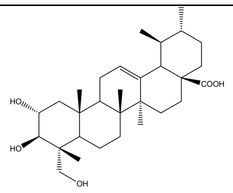
DB56	Docosanoyl (*E*)-ferulate	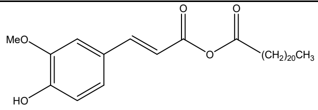
DB57	Tetracosyl (*Z*)-*p*-coumarate	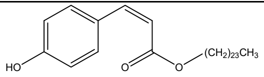
DB58	Tetracosyl (*E*)-*p*-coumarate	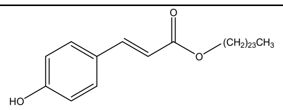
DB59	trans-Tetracosylferulate [(E)-Ferulic acid tetracosyl ester]	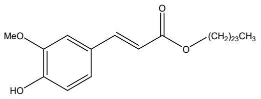
DB60	cis-Docosylferulate	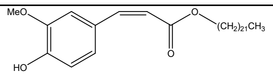
DB61	(*Z*)-Ferulic acid tetracosyl ester	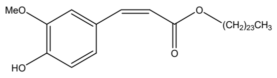
DB62	*n*-Eicosyl *trans*-ferulate	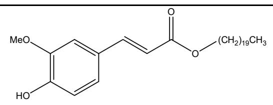
DB63	n-Docosyl 4-hydroxy-trans-cinnamate	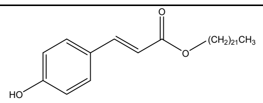
DB64	2-(p-Hydroxyphenyl) ethyl p-coumarate	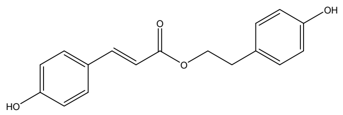
DB65	*p*-Hydroxybenzoic acid	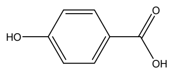
DB66	p-Hydroxybenzaldehyde	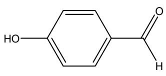

Table S1. The structures of 83 compounds from Dendrobium sp. docked against SARS-CoV-2 spike protein (cont.)

Code	Compound	Structure
DB67	Ferulic acid	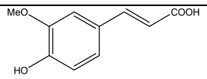
DB68	Dihydroconiferyl dihydro-p-coumarate	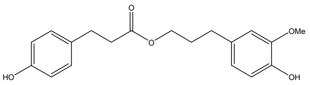
DB69	Methyl haematommate	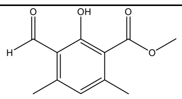
DB70	Methyl 2,4-dihydroxy-3,6-dimethylbenzoate	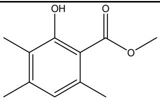
DB71	Vanillin	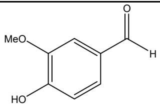
DB72	Coniferyl aldehyde	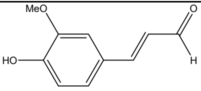
DB73	RF-3192C	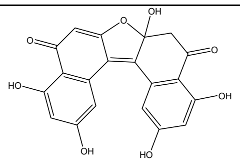
DB74	(-)-Shikimic acid	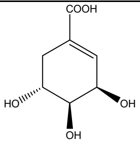
DB75	Diorcinolic acid	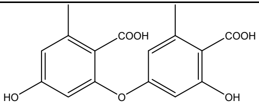
DB76	Dendrofalconerol A	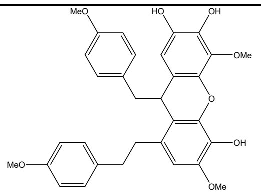
DB77	Dendrofalconerol B	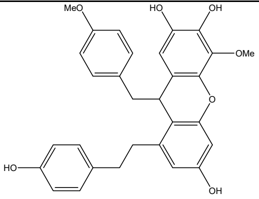

Table S1. The structures of 83 compounds from Dendrobium sp. docked against SARS-CoV-2 spike protein (cont.)

Code	Compound	Structure
DB78	Dendroscabrol B	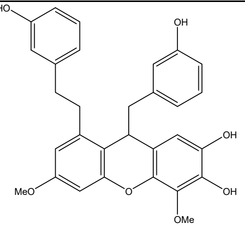
DB79	Dendrosignatol	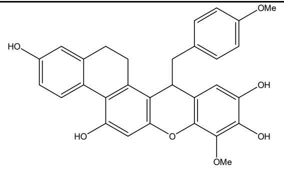
DB80	(−)-Dendroparishiol	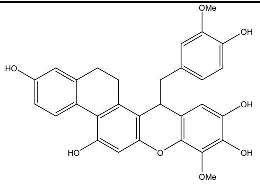
DB81	Phoyunnanin E	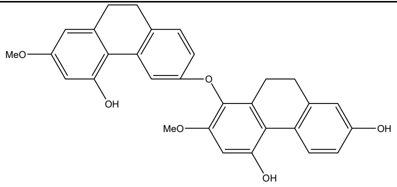
DB82	Phoyunnanin C	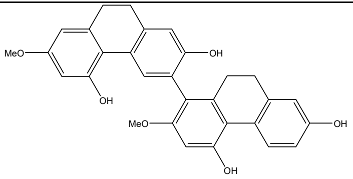
DB83	Dendropalpebrone	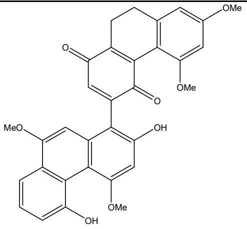

3. Discussion

The binding modes of DB31, DB40 and DB51 with spike protein were shown in Figure S9.

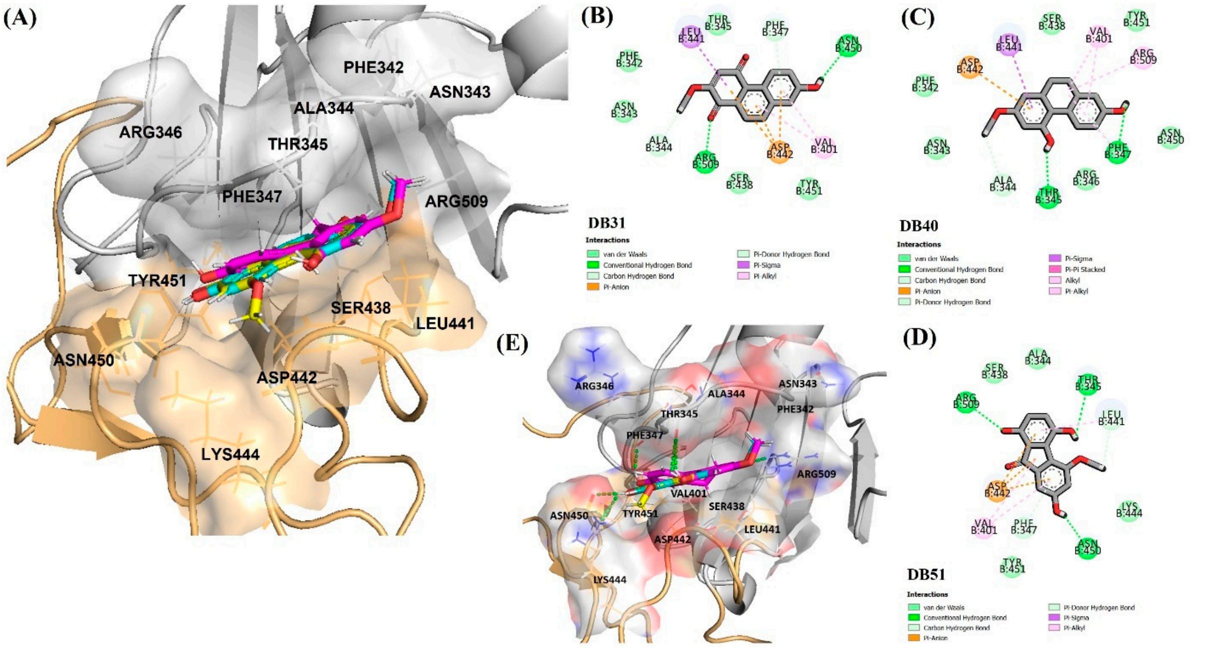

Figure S1. Binding modes of DB31, DB40 and DB51 located in the S1-RBD of spike protein adjacent to the binding motif (RBM) region in an orange color surface; (A) surface view of DB31, DB40 and DB51 binding position between RBM (orange color) and RBD (gray color) regions.; (B) amino acid interactions of DB31 (B), DB40 (C), DB51 (D) and (E) surface view (color by atom type) of DB31, DB40 and DB51 binding modes (E).
